# Case report: Challenges in the diagnosis of Eosinophilic esophagitis

**DOI:** 10.5339/qmj.2023.sqac.30

**Published:** 2023-11-26

**Authors:** Jassim Ahmed Khan, Tayseer Ibrahim, Maryam Ali Al-Nesf

**Affiliations:** ^1^Allergy and Immunology Division, Department of Medicine, Hamad Medical Corporation, Doha Qatar Email: drjassimahmedkhan.2021@gmail.com

## Introduction

Diagnosing Eosinophilic esophagitis (EoE) has been challenging as the clinical presentation is often indeterminate as symptoms resemble GERD, achalasia, Celiac, Crohns, Hypereosinophilic syndrome, Eosinophilic GI disease, and drug hypersensitivity. Eosinophilic esophagitis (EoE) is a chronic immune-mediated inflammatory disorder characterized by eosinophilic infiltration of the esophagus strongly correlated with chronic seasonal allergies, atopy, asthma, and asthma eczemas in children and adults having male preponderance. It has a prevalence of approximately 34.4/100 000 worldwide. A Case report is presented below with clinical, pathological, and endoscopic findings of (EoE).

## Case Report

A 34-year-old Male with a childhood history of Bronchial asthma was referred from Gastroenterology with a presumed diagnosis of GERD, unresponsive to 8 weeks of Pantoprazole 40mg BID to the Allergy and Immunology department for further evaluation for complaints of generalized pruritus since two days, persistent dysphagia and upper abdominal discomfort since three months exacerbated by egg meals and relieves by avoidance of it. Persistent dysphagia was more to solids with dull epigastric pain. The patient denied a history of urticarial rashes, flushing, vomiting, itching/redness of eyes, cough, chest pain tightness, chest tightness, and dyspnoea. The patient was compliant with Albuterol MDI (90 mcg, two inhalations/day), has no recent exacerbations, and regularly follows up in the asthma clinic. His Blood investigations were unremarkable, and the skin prick test was negative for common allergens. The upper GI endoscopy showed stacked oesophageal rings, linear furrows, and proximal and distal sections of the biopsy showed mucosal eosinophilic infiltration (>15 Eosinophils HPF) and eosinophil micro-abscesses. A diagnosis of Eosinophilic esophagitis was made. The patient was prescribed Topical Fluticasone (220 mcg/spray, four sprays daily in divided doses) and Esomeprazole 40mg once daily for eight weeks with dietary modifications. Symptoms improved, and subsequently, the patient was started with maintenance therapy Budesonide (0.5mg twice daily).

## Conclusion

Misdiagnosis of EoE as GERD leads to prolonged PPI treatment with no improvement. A definitive diagnosis of EoE should be strongly considered after correlating allergic history, clinical symptoms, and positive investigatory findings, which can result in better disease and treatment outcomes.

